# Predictors of treatment outcomes in adults with drug–sensitive Tuberculosis in Maharashtra, India: A retrospective study

**DOI:** 10.1371/journal.pgph.0006539

**Published:** 2026-11-06

**Authors:** Raghavan Parthasarathy, Yashi Raj, Nabanita Majumder, Mithun Mitra, Sarika Mehra, Raghuram Rao, Shobini Rajan

**Affiliations:** 1 National Disease Modelling Consortium, Indian Institute of Technology, Mumbai, India; 2 Department of Physics, Indian Institute of Technology, Mumbai, India; 3 Department of Chemical Engineering, Indian Institute of Technology, Mumbai, India; 4 Central Tuberculosis (TB) Division, National TB Elimination Programme, Ministry of Health and Family Welfare, Government of India, New Delhi, India; Dr D Y Patil Medical College Hospital and Research Centre, INDIA

## Abstract

Tuberculosis (TB) remains the leading infectious cause of death worldwide, with India accounting for nearly one-fourth of global TB cases. Ni-kshay, the country’s digital case-based TB notification platform is rich in data pertaining to the continuum of care of TB patients. This study aims to develop a standardized analytical approach to programmatic data to identify predictors of unfavourable treatment outcomes and mortality among adult drug-sensitive TB patients at the state level for Maharashtra during 2021 and 2022. Two separate analyses were undertaken comparing treatment success with: (1) unfavourable outcomes (death, treatment failure, loss to follow-up, regimen change, or not evaluated); and (2) mortality. Age group, gender and pre-treatment weight were entered together in a single logistic regression model, and each remaining risk factor was entered into a separate model alongside those three covariates, to compute adjusted odds ratios (aOR). Records with no entry for diabetes, HIV, tobacco or alcohol were retained as a “not recorded” or “unknown” category. Collinearity across variables was assessed using Cramer’s V and variance inflation factors. The final cohort included 323,124 cases for unfavourable outcome analysis and 315,579 cases for mortality analysis. Increasing age, male gender, lower body weight, known HIV and diabetes comorbidities, tobacco and alcohol consumption, and “unknown” status for behavioural risks and comorbidity status were significantly associated with increased odds of both unfavourable outcomes and mortality. This study highlights the utility of programmatic data in identifying high-risk TB patients and offers a reproducible analytic framework.

## Introduction

Tuberculosis (TB) continues to be the leading cause of mortality among infectious diseases. India has the highest TB burden globally, accounting for nearly one-fourth of the world’s TB cases. As per the Global TB Report 2025 the estimated number of TB-related deaths in India in 2024 was 305,041 (234,696–379,969), with a case fatality ratio (CFR) of 11% (8–15%) [1]. This reflects a reduction of over ~28% in terms of the TB deaths, against the interim milestone for 2025 as per the End TB strategy [2].

Considering this gap to end TB, the Government of India had prioritised TB elimination through its National Strategic Plan (NSP), which outlines the targets required to be achieved and the timelines for ending TB as a public health problem in the country [3]. Central to the NSP is the National TB Elimination Programme (NTEP), formerly known as the Revised National Tuberculosis Control Programme (RNTCP), which ensures the provision of free diagnostic and treatment services nationwide. In support of effective surveillance and programmatic monitoring, the Government of India introduced the Ni-kshay platform in 2012, a digital, case-based TB notification and management system covering both public and private health sectors.

Ni-kshay, means “end (*Ni*) tuberculosis (*kshay*),” symbolises India’s commitment to defeat tuberculosis (TB). The evolution of Ni-kshay has coincided with expanded programmatic support measures, such as the introduction of the Ni-kshay Poshan Yojana (NPY), launched in 2018, which provides TB patients with INR 1000 per month to mitigate nutrition-related vulnerabilities, Pradhan Mantri TB Mukt Bharat Abhiyaan (PMTBMA, 2022) where TB patients in need are linked with community supporters willing to provide nutritional support to the affected households through Nikshay Mitras [4,5]. Ni-kshay has evolved into a repository of rich, patient-level data encompassing clinical, demographic, and treatment-related variables across India’s vast and diverse populations [6].

Programmatically, treatment success is a key indicator for monitoring the effectiveness of TB treatment services and tracking progress towards the targets set under the End TB Strategy, including the reduction of TB-related mortality [7]. According to the India TB Report 2024, across the country, ~ 6.2% of TB patients experienced unfavourable outcomes (i.e., deaths: 3.6%, loss-to-follow-up:2.1%, treatment failure:0.5%) [8]. Understanding and addressing the determinants of these outcomes is critical for meeting NSP’s vision.

This is a retrospective observational study focused on adult patients (≥15 years of age) diagnosed with drug-sensitive TB (DS-TB) and treated in Maharashtra, a high-TB burden state, during the years 2021 and 2022. The study aims to systematically analyse Ni-kshay data to identify predictors of unfavourable treatment outcomes as well as mortality using a robust, reproducible statistical framework. Through this effort, we aim to provide a replicable model including a reporting framework for Ni-kshay-based statistical analysis.

## Methodology

### Ethics statement

This study used anonymised, non–patient-identifiable secondary data from the programmatic database. The data were received under the memorandum of understanding between IIT Bombay (NDMC) and the Ministry of Health and Family Welfare (MoHFW), Government of India, as part of routine modelling support to the National TB Elimination Programme. Before undertaking the study, ethical approval for the work was obtained from the Institutional Review Board (IRB) at IIT Bombay (IRB-2025–028_SM_M). The analysis was also conducted with administrative approval from the Central TB Division, NTEP, Government of India. The manuscript was prepared in accordance with the Strengthening the Reporting of Observational Studies in Epidemiology (STROBE) statement.

### General setting and data source

This was a collaborative initiative between the National Disease Modelling Consortium (NDMC), at the Indian Institute of Technology, Bombay (IIT-B) [9] and the Central TB Division (CTD), Government of India (GoI). This was a retrospective observational study utilizing routinely collected programmatic data to standardize the procedure for understanding the determinants of unfavourable treatment outcomes and mortality among adult (≥15 years) DS-TB patients from Maharashtra in 2021 and 2022.

The state of Maharashtra was purposively selected for this analysis in consultation with the Central TB Division (CTD), based on its high tuberculosis (TB) burden. The extraction methodology from Ni-kshay applied filters based on “diagnosing facility” and “notification date” for the current case study. To focus the analysis on treatment outcomes, only cases with documented treatment initiation were included. Specifically, records lacking a valid entry under the “spectrum treatment initiation date” variable, either blank or marked as “NA”, were excluded from the study cohort, as these represented individuals not initiated on treatment. Given the longer and more complex treatment timelines for drug-resistant TB (DR-TB), this study was limited to patients diagnosed with drug-sensitive TB (DS-TB). The objective was to assess determinants of treatment outcomes within a programmatic cohort for whom standard treatment timelines and outcome measures apply within a span of 12 months. It was decided to use only the notification register and comorbidity register in Ni-kshay as the primary data-source, considering the relevance for building the case study (data was accessed on 21/08/2025). The study focused on cohorts from 2021 and 2022, as these represent completed treatment cycle cohorts with outcomes available for analysis as well as in accordance with national programmatic guidance, which recommends outcome evaluation 12 months after diagnosis. Cohorts from 2023 and 2024 were excluded, as treatment for a substantial proportion of these cases was either ongoing or not yet eligible for outcome assessment [7].

### Specific setting and definitions

Two outcome-based comparisons were conducted (1) treatment success versus unfavourable TB treatment outcomes and (2) treatment success versus mortality. Treatment success was defined as a final outcome of “cured” or “treatment completed.” Mortality referred to outcomes marked as “died.” Unfavourable outcomes included “died,” “loss to follow-up,” “failure,” “treatment regimen changed,” and “not evaluated.” Recently, countries and their research programmes have begun to categorise “not evaluated” under “unfavourable outcomes” [10–12]. To align with NTEP definitions [7], we also be conducted a sensitivity analysis, excluding those individuals with the outcome of “not evaluated” from the unfavourable outcomes cohort. The counting of the number of cases was done using the variable “*instance*”.

### Risk factor/determinants

The risk-factors considered for analysis considering the completeness and correctness of the information captured were as follows: demographic variables: age (>15 years), gender; anthropometric variable for capturing nutritional status: weight (Kg); TB related – site of disease (pulmonary or extra-pulmonary); clinical comorbidities: diabetes status, HIV status; and behavioural risk factors: tobacco consumption status and alcohol consumption status. The risk factors that were available as integers were categorized and those which were non-numerical were recoded for analysis. Height was not utilized because of the non-availability of the information in more than 1/3^rd^ of the records. To ensure completeness in the dataset, those cases where weight was not available, were excluded from analysis.

### Analytical procedure including data pre-processing

The comorbidity register was merged with the notification register by matching using the variable “instance”. After merging, the cases having age less than 15 years and drug-resistant TB cases (*DRTB case definition: cases with a value of “1” in the variable “RR resistance detected” or “INH resistance detected”)* were excluded. Pairwise association between categorical variables was quantified using Cramér’s V, computed for all 28 pairs of the eight predictors. Collinearity was additionally assessed formally using variance inflation factors, reported for each of the models fitted in the primary analysis. Values above 5 were taken to warrant attention of collinearity [13]. Univariate and multivariate logistic regression analysis, using STATA version 19 (StataCorp. 2025. *Stata Statistical Software: Release 19*. College Station, TX: StataCorp LLC) was done to report the risks of various factors and reported as “odds ratio (OR)” and “adjusted odds ratio (aOR)”, respectively, with 95% confidence intervals. In the adjusted analysis, age group, gender and pre-treatment body weight were entered together in a single model and are therefore mutually adjusted for one another. Each of the remaining risk factors, namely diabetes status, HIV status, current tobacco use, history of alcohol intake and site of disease, was entered into a separate model containing that variable together with age group, gender and pre-treatment weight. Reference categories were fixed a priori and were 15–29 years for age group, female for gender, 61–80 kg for pre-treatment weight, non-diabetic, HIV negative, no current tobacco use, no history of alcohol intake and pulmonary disease for site; they are mentioned explicitly in Tables 1 and 2.

**Table 1:** Descriptive and inferential analysis on the risk factor for unfavourable treatment outcomes from Ni-kshay for Maharashtra (2021 and 2022).

Variable and category	Treatment success (n = 301,318)	Unfavourable outcome (n = 21,806)	Total (n = 323,124)	p value	OR	95% CI	aOR	95% CI
**Age group**				<0.001				
**15 to 29 years (reference)**	104444 (34.7)	3692 (16.9)	108136 (33.5)		**1.00**	**Reference**	**1.00**	**Reference**
30 to 44 years	83354 (27.7)	5423 (24.9)	88777 (27.5)		1.84	1.76 to 1.92	1.90	1.82 to 1.98
45 to 59 years	62086 (20.6)	5649 (25.9)	67735 (21.0)		2.57	2.47 to 2.69	2.64	2.52 to 2.75
60 years and above	51434 (17.1)	7042 (32.3)	58476 (18.1)		3.87	3.72 to 4.04	3.85	3.69 to 4.01
**Gender**				<0.001				
**Female (reference)**	138824 (46.0)	7514 (34.5)	146338 (45.3)		**1.00**	**Reference**	**1.00**	**Reference**
Male	162338 (53.9)	14271 (65.4)	176609 (54.7)		1.62	1.58 to 1.67	1.50	1.46 to 1.55
Transgender	156 (0.1)	21 (0.1)	177 (0.1)		2.49	1.58 to 3.92	2.52	1.59 to 4.0
**Pre-treatment body weight**				<0.001				
Greater than 80 kg	4127 (1.4)	238 (1.1)	4365 (1.4)		0.97	0.85 to 1.11	1.02	0.89 to 1.18
**61 to 80 kg (reference)**	34549 (11.5)	2052 (9.4)	36601 (11.3)		**1.00**	**Reference**	**1.00**	**Reference**
41 to 60 kg	182738 (60.6)	11910 (54.6)	194648 (60.2)		1.10	1.05 to 1.15	1.21	1.16 to 1.27
21 to 40 kg	79219 (26.3)	7511 (34.4)	86730 (26.8)		1.60	1.52 to 1.68	2.14	2.03 to 2.25
20 kg or less	685 (0.2)	95 (0.4)	780 (0.2)		2.34	1.88 to 2.91	3.29	2.63 to 4.11
**Diabetes status**				<0.001				
**Non-diabetic (reference)**	277133 (92.0)	18525 (85.0)	295658 (91.5)		**1.00**	**Reference**	**1.00**	**Reference**
Diabetic	17865 (5.9)	2181 (10.0)	20046 (6.2)		1.83	1.74 to 1.91	1.43	1.36 to 1.50
Not recorded/unknown	6320 (2.1)	1100 (5.0)	7420 (2.3)		2.60	2.44 to 2.78	2.57	2.40 to 2.74
**HIV status**				<0.001				
**HIV negative (reference)**	291380 (96.7)	19705 (90.4)	311085 (96.3)		**1.00**	**Reference**	**1.00**	**Reference**
Living with HIV	8216 (2.7)	1708 (7.8)	9924 (3.1)		3.07	2.91 to 3.24	2.84	2.69 to 3.00
Not recorded/unknown	1722 (0.6)	393 (1.8)	2115 (0.7)		3.37	3.02 to 3.77	3.48	3.10 to 3.89
**Current tobacco use**				<0.001				
**No current tobacco use (reference)**	228091 (75.7)	14849 (68.1)	242940 (75.2)		**1.00**	**Reference**	**1.00**	**Reference**
Yes	18966 (6.3)	2026 (9.3)	20992 (6.5)		1.64	1.56 to 1.72	1.20	1.14 to 1.26
Not recorded/unknown	54261 (18.0)	4931 (22.6)	59192 (18.3)		1.40	1.35 to 1.44	1.43	1.38 to 1.47
**History of alcohol intake**				<0.001				
**No history of alcohol intake (reference)**	231557 (76.8)	14981 (68.7)	246538 (76.3)		**1.00**	**Reference**	**1.00**	**Reference**
Yes	10273 (3.4)	1444 (6.6)	11717 (3.6)		2.17	2.05 to 2.30	1.67	1.57 to 1.77
Not recorded/unknown	59488 (19.7)	5381 (24.7)	64869 (20.1)		1.40	1.35 to 1.44	1.44	1.39 to 1.49
**Site of disease**				<0.001				
**Pulmonary (reference)**	191739 (63.6)	15417 (70.7)	207156 (64.1)		**1.00**	**Reference**	**1.00**	**Reference**
Extra-pulmonary	109579 (36.4)	6389 (29.3)	115968 (35.9)		0.73	0.70 to 0.75	0.95	0.92 to 0.98

OR-Odds ratio, aOR – adjusted odds ratio, CI – confidence interval

**Table 2:** Descriptive and inferential analysis on the risk factor for TB mortality from Ni-kshay for Maharashtra (2021 and 2022).

Variable and category	Treatment success (n = 301,318)	Died (n = 14,261)	Total (n = 315,579)	p value	OR	95% CI	aOR	95% CI
**Age group**				<0.001				
**15 to 29 years (reference)**	104444 (34.7)	1504 (10.5)	105948 (33.6)		**1.00**	**Reference**	**1.00**	**Reference**
30 to 44 years	83354 (27.7)	3062 (21.5)	86416 (27.4)		2.55	2.40 to 2.72	2.72	2.56 to 2.90
45 to 59 years	62086 (20.6)	3904 (27.4)	65990 (20.9)		4.37	4.11 to 4.64	4.67	4.39 to 4.96
60 years and above	51434 (17.1)	5791 (40.6)	57225 (18.1)		7.82	7.38 to 8.28	8.06	7.60 to 8.54
**Gender**				<0.001				
**Female (reference)**	138824 (46.0)	4808 (33.7)	143632 (45.5)		**1.00**	**Reference**	**1.00**	**Reference**
Male	162338 (53.9)	9439 (66.2)	171777 (54.4)		1.68	1.62 to 1.74	1.47	1.41 to 1.52
Transgender	156 (0.1)	14 (0.1)	170 (0.1)		2.59	1.50 to 4.48	2.63	1.51 to 4.61
**Pre-treatment body weight**				<0.001				
Greater than 80 kg	4127 (1.4)	134 (0.9)	4261 (1.4)		0.92	0.77 to 1.10	1.00	0.83 to 1.20
**61 to 80 kg (reference)**	34549 (11.5)	1223 (8.6)	35772 (11.3)		**1.00**	**Reference**	**1.00**	**Reference**
41 to 60 kg	182738 (60.6)	7497 (52.6)	190235 (60.3)		1.16	1.09 to 1.23	1.31	1.23 to 1.40
21 to 40 kg	79219 (26.3)	5325 (37.3)	84544 (26.8)		1.90	1.78 to 2.02	2.72	2.55 to 2.91
20 kg or less	685 (0.2)	82 (0.6)	767 (0.2)		3.38	2.67 to 4.28	5.34	4.18 to 6.82
**Diabetes status**				<0.001				
**Non-diabetic (reference)**	277133 (92.0)	11881 (83.3)	289014 (91.6)		**1.00**	**Reference**	**1.00**	**Reference**
Diabetic	17865 (5.9)	1660 (11.6)	19525 (6.2)		2.17	2.05 to 2.29	1.56	1.48 to 1.65
Not recorded/unknown	6320 (2.1)	720 (5.0)	7040 (2.2)		2.66	2.45 to 2.88	2.61	2.41 to 2.84
**HIV status**				<0.001				
**HIV negative (reference)**	291380 (96.7)	12712 (89.1)	304092 (96.4)		**1.00**	**Reference**	**1.00**	**Reference**
Living with HIV	8216 (2.7)	1285 (9.0)	9501 (3.0)		3.59	3.37 to 3.81	3.40	3.19 to 3.63
Not recorded/unknown	1722 (0.6)	264 (1.9)	1986 (0.6)		3.51	3.08 to 4.01	3.68	3.22 to 4.22
**Current tobacco use**				<0.001				
**No current tobacco use (reference)**	228091 (75.7)	9721 (68.2)	237812 (75.4)		**1.00**	**Reference**	**1.00**	**Reference**
Yes	18966 (6.3)	1308 (9.2)	20274 (6.4)		1.62	1.52 to 1.72	1.11	1.04 to 1.18
Not recorded/unknown	54261 (18.0)	3232 (22.7)	57493 (18.2)		1.40	1.34 to 1.46	1.45	1.39 to 1.51
**History of alcohol intake**				<0.001				
**No history of alcohol intake (reference)**	231557 (76.8)	9827 (68.9)	241384 (76.5)		**1.00**	**Reference**	**1.00**	**Reference**
Yes	10273 (3.4)	914 (6.4)	11187 (3.5)		2.10	1.95 to 2.25	1.57	1.46 to 1.69
Not recorded/unknown	59488 (19.7)	3520 (24.7)	63008 (20.0)		1.39	1.34 to 1.45	1.46	1.40 to 1.52
**Site of disease**				<0.001				
**Pulmonary (reference)**	191739 (63.6)	9962 (69.9)	201701 (63.9)		**1.00**	**Reference**	**1.00**	**Reference**
Extra-pulmonary	109579 (36.4)	4299 (30.1)	113878 (36.1)		0.76	0.73 to 0.78	1.10	1.05 to 1.14

OR-Odds ratio, aOR – adjusted odds ratio, CI – confidence interval

### Handling of records with no recorded status

For diabetes status, HIV status, tobacco use and alcohol intake, records with no entry were coded as “not recorded” or “unknown” and retained in the analysis rather than excluded. A missing entry in Ni-kshay is not a clinical state rather it records whether the particular public health action measure or service was provided to the beneficiary and captured or not. Two distinct quantities therefore arise and were treated separately. The coefficient on the not-recorded category was interpreted as the excess risk carried by a record for which no assessment was documented, which is a health-system indicator. The coefficients on the recorded categories were interpreted as the effect of the underlying clinical or behavioural exposure. Because retaining a missing-indicator category could lead to selection bias, two model specifications were compared as part of sensitivity analysis in the regression model: Model A, the missing-indicator as a separate sub-category within each of the factors was fitted on the whole cohort, and Model B, restricted to only patients with a status recorded or known for all four variables.

## Results

The final dataset included in the analysis comprised TB cases notified in Maharashtra between 2021 and 2022. Following exclusions based on predefined criteria (see Fig 1), 323,124 cases were included in the final cohort for the analysis of unfavourable treatment outcomes, and 315,579 cases were analysed for TB-related mortality (excluding cases with outcomes other than “DIED” from the unfavourable outcomes).

**Fig 1:**
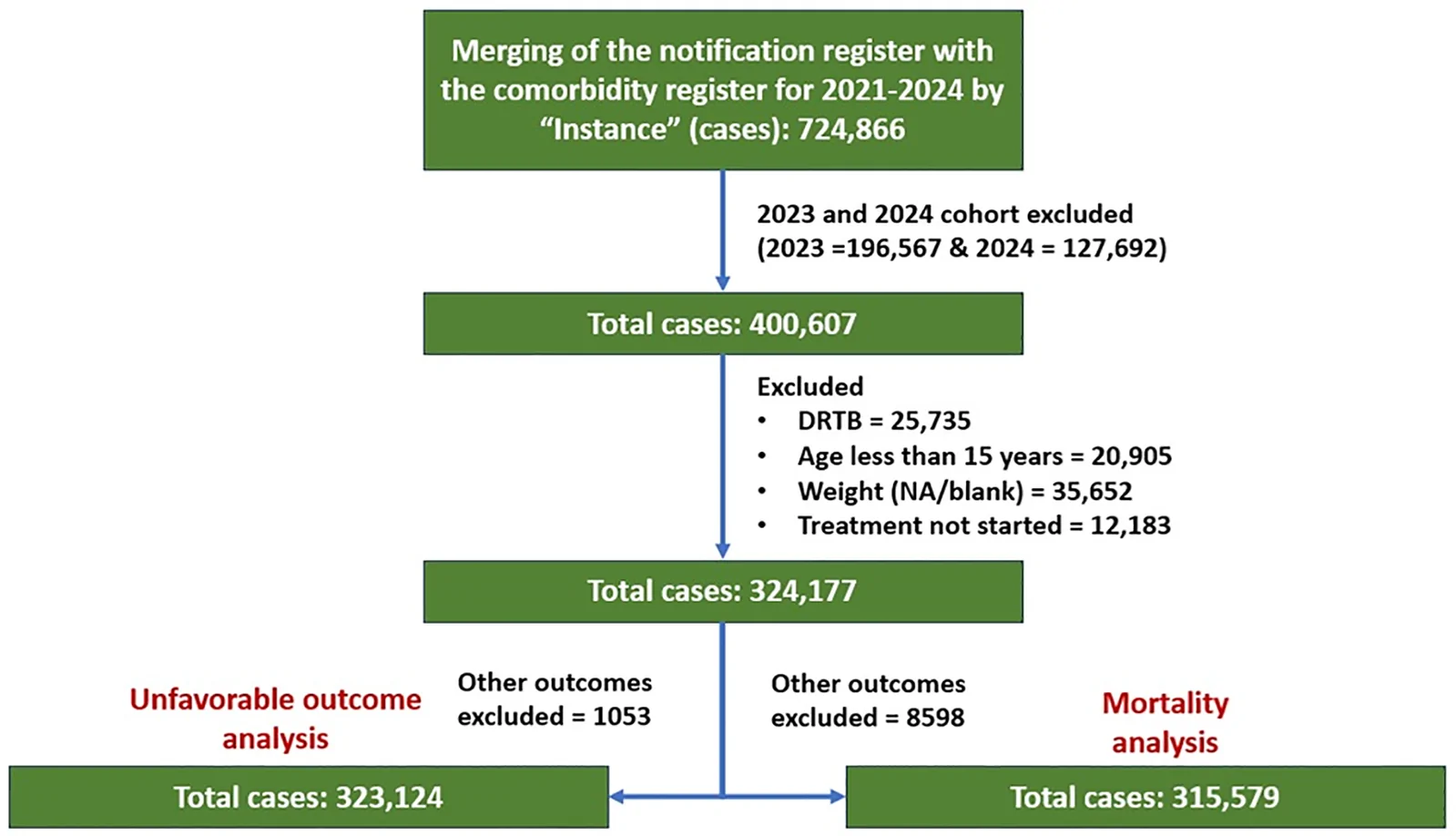
DRTB - drug resistant TB, NA - not available or recorded. Mortality referred to outcomes marked as “died.” Unfavourable outcomes included “died,” “loss to follow-up,” “failure,” “treatment regimen changed,” and “not evaluated”.

### Demographic Characteristics (Table 1 & 2; Fig 2)

The mean (SD) age of patients in the unfavourable outcomes cohort was 48.9 (17.9) years, and 53.1 (17.4) years in the mortality cohort. The burden of both unfavourable outcomes and mortality was markedly higher among individuals aged ≥60 years, who accounted for 32.3% of all unfavourable outcomes and 40.6% of all deaths. Similarly, males were disproportionately affected, comprising 65.4% of the unfavourable outcome group and 66.2% of deaths. Multivariate logistic regression analysis indicated a consistent and significant increase in risk with advancing age. Patients aged more than 60 years had the highest adjusted odds ratios (aORs) for both outcomes: 3.85 (95% CI: 3.69–4.01) for unfavourable outcomes and 8.06 (95% CI: 7.60–8.54) for mortality. Gender-based disparities were evident; males had elevated risk (aOR: 1.50 [1.46–1.55] for unfavourable outcomes; 1.47 [1.41–1.52] for mortality), and transgender individuals demonstrated substantially higher risk (aOR: 2.52 [1.59–4.00] for unfavourable outcomes; 2.63 [1.51–4.61] for mortality). Considering the lesser number of patients who were transgender, this was not emphasized for statistical reasons.

**Fig 2:**
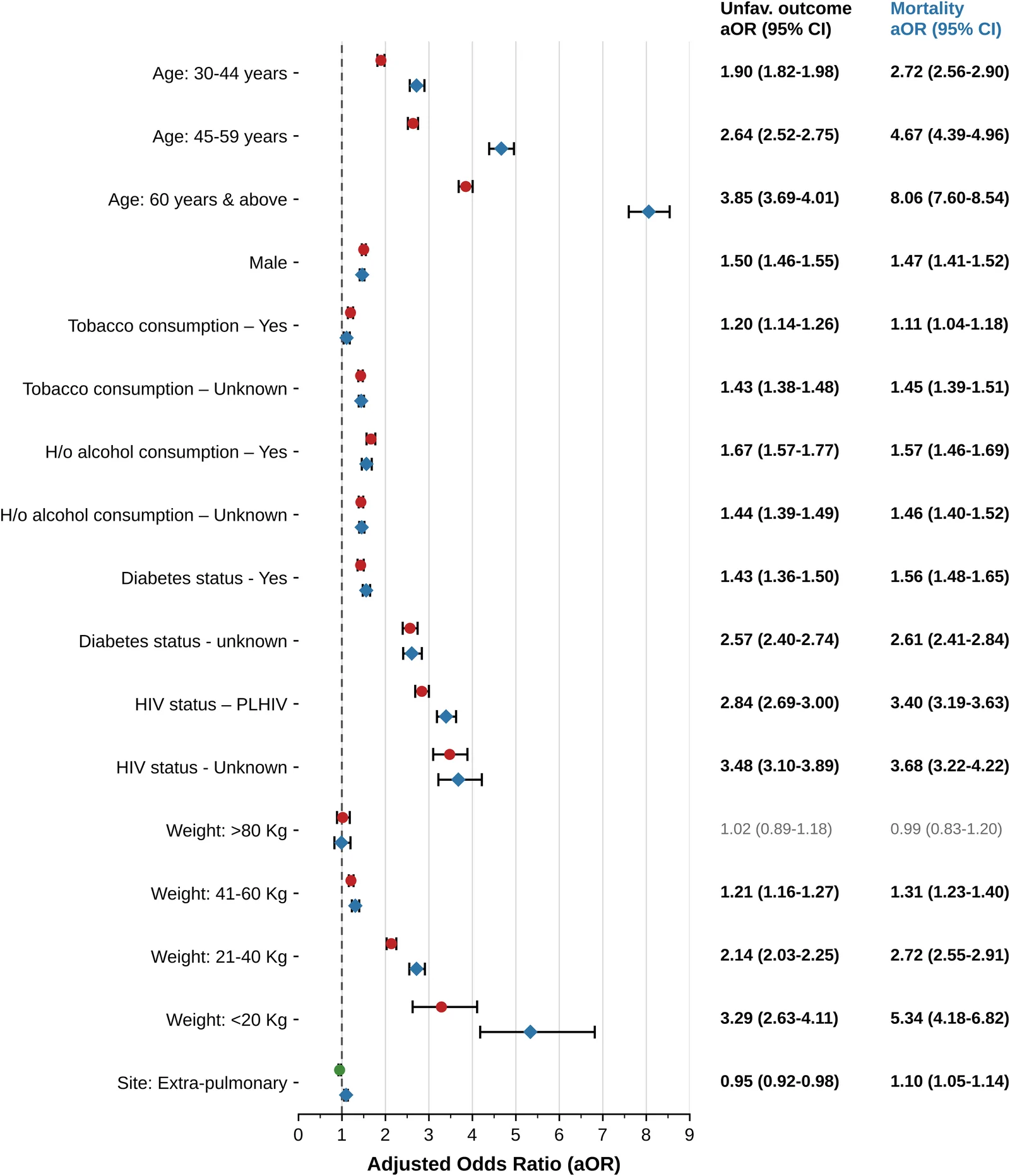
Risk factors associated with unfavourable outcomes and mortality, among drug sensitive TB patients. Legends: aOR: adjusted odds ratio, unfav. outcomes - unfavourable outcomes, PLHIV - People Living with Human immunodeficiency virus infection, Site - site of TB disease, H/o - self reported history.

### Completeness of baseline assessment (Supporting information: S10 Table)

Baseline status was recorded for all four comorbidity and behavioural variables in 253,575 of 323,124 patients (78.5%) in the unfavourable outcome cohort and in 248,027 of 315,579 (78.6%) in the mortality cohort. Non-recording was almost entirely confined to the behavioural variables: diabetes status was unrecorded for 7,420 patients (2.3%) and HIV status for 2,115 (0.7%), against 59,192 (18.3%) for current tobacco use and 64,869 (20.1%) for history of alcohol intake. Non-recording of the two behavioural variables was highly concordant: of the 59,192 records with no tobacco entry, 54,989 (92.9%) also had no alcohol entry. The same pattern of 92.9% was obtained in the mortality cohort.

### Behavioural risk factors (Tables 1 and 2; Fig 2)

Both tobacco and alcohol consumption were associated with unfavourable outcomes as well as mortality. Tobacco users accounted for 9.3% of the unfavourable outcome cohort and 9.2% of the mortality cohort. Alcohol consumption was reported in 6.6% and 6.4% of these respective cohorts. Notably, the unknown status for both tobacco (22.5% for unfavourable outcomes; 22.6% for mortality) and alcohol consumption (24.7% for both outcomes) remained high. Among categorical variables, Cramér’s V for the association between current tobacco use and history of alcohol intake was 0.713 in the unfavourable outcome cohort and 0.712 in the mortality cohort (Supporting information: S5 Table). This figure is largely attributable to concordant non-recording rather than to co-occurring behaviour, since 92.9% of records with an unrecorded tobacco status also had an unrecorded alcohol status. The largest variance inflation factor in any primary model was 2.675 (less than 5) (Supporting information: S8 Table). Both variables were therefore retained separately, aside from their VIF’s being lesser and given their independent biological effects on TB pathogenesis as well as the different programmatic responses they call for. Regression analyses showed a higher risk associated with alcohol consumption (aOR: 1.67 [1.57–1.77]) for unfavourable outcomes; 1.57 [1.46–1.69] for mortality than with tobacco consumption. While known tobacco consumption was linked to elevated risk, those with unknown tobacco status had slightly higher aORs (1.43 [1.38–1.48] for unfavourable outcomes; 1.45 [1.39–1.51] for mortality).

### Comorbidities and anthropometry (Tables 1 and 2; Fig 2)

Among the cases, diabetes was reported in 10% of those with unfavourable outcomes and 11.6% of those who died. PLHIV constituted 7.8% and 9% of the respective groups. Both diabetes and HIV status were significant predictors of unfavourable outcomes, even after adjusting for demographic variables and weight. Individuals with unknown HIV or diabetes status also exhibited increased risk. Lower body weight emerged as a significant predictor of both unfavourable treatment outcomes and mortality among TB patients. Individuals in the 41–60 kg weight category constituted the majority of those with unfavourable outcomes, representing 54.6% of the unfavourable outcome group and 52.6% of those who died. A clear dose-response relationship was observed, with the adjusted odds ratio (aOR) for adverse outcomes increasing as body weight declined. Patients weighing less than 40 kg were found to be at the highest risk, indicating a strong vulnerability. An analysis stratified by age and weight revealed distinct shifts in the demographic and clinical profile across outcome categories (Supporting information: S4 Table). The cohort with successful treatment outcomes was predominantly composed of younger to middle-aged individuals (15–59 years) within the 41–60 kg weight range. However, in the group with unfavourable outcomes, there was a marked increase in the proportion of older adults (≥60 years), who accounted for 32.3% of this subgroup, along with a higher prevalence of individuals in the severely low weight range (21–40 kg). This pattern was even more pronounced in the mortality cohort. Patients aged 60 years and above contributed to over 40% of all recorded deaths, while more than 90% of the deaths occurred among those weighing less than 60 kg. These findings highlight a synergistic and compounding risk posed by advanced age and low body weight, underscoring their critical role in the transition from treatment success to unfavourable outcomes.

### TB disease site and outcome relationship (Tables 1 and 2; Fig 2)

Cases with extra-pulmonary TB (EPTB) demonstrated lower proportions of both unfavourable outcomes and mortality. Interestingly, multivariate analysis revealed a divergent trend where EPTB was protective against unfavourable outcomes but conferred an increased risk of mortality. The temporal trends across 2021–2022 remained largely stable (Supporting information: S1 Table, S2 Table and S3 Table).

### Sensitivity analysis

Three sensitivity analyses were carried out to ensure the robustness of risk factor associations for unfavourable treatment outcomes as well as mortality. First, we compared baseline characteristics between patients with complete versus missing weight. Second, we performed multiple imputation using predictive mean matching with 5 nearest neighbours (k = 5) to assess the impact of missing weight data on association (aOR) estimates. We generated 10 imputed datasets using age group, gender, diabetes status, HIV status, substance consumption, and site of TB disease as predictors). Third, we excluded all patients classified as ‘not evaluated’ (programmatic outcome, auto-assigned for cases when no treatment outcome gets assigned after the expected end date of treatment after 6 months) from the unfavourable outcome category and repeated the overall multivariable analysis.

Missing data versus complete case comparison revealed that ~6% of patients lacked weight information with similar distribution of variables between the two groups (*Supporting information:* S6a Table and S6b Table). Multiple imputation using predictive mean matching produced consistent results across all 10 imputations (both in the unfavourable outcome and mortality comparison), with no impossible values generated. In multivariable models, for unfavourable outcomes and mortality the aOR for factors showed consistent associations across methods, indicating that missing weight data did not substantially bias findings (*Supporting information:* S7 Table) as compared to the primary analysis (Tables 1 and 2). Finally, upon exclusion of all patients classified as ‘not evaluated’ from the unfavourable outcome category and repeating the overall analysis, the aORs for all risk factors remained virtually unchanged (not shown), confirming that this category (n = 513) in the current unfavourable outcomes cohort did not affect the observed associations.

### Addressing the unknown or the not-recorded category (Supporting information: S9a Table and S9b Table)

Restricting the analysis to the 253,575 patients with a status recorded for all four variables left the adjusted odds ratios for the recorded categories essentially unchanged. For an unfavourable outcome, the adjusted odds ratio for diabetes was 1.43 (95% CI 1.36 to 1.50) in the whole cohort and 1.44 (1.36 to 1.52) in the restricted analysis; for HIV infection 2.84 (2.69 to 3.00) and 2.84 (2.66 to 3.03); for current tobacco use 1.20 (1.14 to 1.26) and 1.22 (1.16 to 1.29); and for history of alcohol intake 1.67 (1.57 to 1.77) and 1.67 (1.57 to 1.77). The largest departure across all recorded categories was 1.8%. The mortality cohort showed similar pattern, with a largest departure of 2.1%.

## Discussion

This study represents one of the first large-scale attempts to apply a standardized analytical protocol to programmatic data for the retrospective analysis of unfavourable treatment outcomes and mortality among adult drug-sensitive TB (DS-TB) patients. By using routine programmatic data from Maharashtra, a high-burden state [8], across two calendar years (2021 and 2022), we demonstrated the value of programmatic data both as a surveillance and decision-support tool when methodologically harnessed. Our analysis affirmed known risk factors [14–19], both from India and worldwide: age, sex, HIV, diabetes and current tobacco and alcohol consumption. Males, older adults (especially above 60), and patients with co-morbidities like diabetes or HIV had higher odds of both unfavourable outcomes and mortality after adjustment. Also, for Maharashtra, we observed that the risk due to HIV was lower than observed across another study in Central India [20], possibly because of the higher coverage of TB-HIV screening and antiretroviral treatment initiation in the region [21].

Notably, the “unknown” status for tobacco, alcohol, diabetes, or HIV [18] emerged as a significant risk category in itself. This finding reinforces the critical importance of high-quality data entry and the need for frontline worker training in holistic risk assessment [22]. From a health systems perspective, the “unknown” category in Ni-kshay could arise from one of several scenarios: (a) the screening test was not offered or not performed at the diagnosing facility; (b) the test was performed but the result was not entered into the Ni-kshay portal, reflecting a data entry gap rather than a clinical gap; (c) the patient was diagnosed and notified by the provider, where integration with NTEP comorbidity screening protocols has historically been weaker; or (d) the patient declined testing due to stigma or logistical barriers. Each of these scenarios represents a distinct point of programmatic challenge that need to be addressed through targeted interventions. This finding aligns with the observations from the assessment of Ni-kshay [6]. However, considering the COVID-19 pandemic and the various constraints within which programmatic services were delivered during 2021 and 2022, including disruptions to routine screening, the completeness of information was understandably lower than what would be expected and should be interpreted with this operational context in mind [8].

A record with no entry in these fields is a record for which the programme did not complete, or did not provide, or did not capture, and such records carried a plausibly higher risk of both an unfavourable outcome and death. Three observations support this reading. First, the adjusted odds ratio for a not-recorded status exceeded that for the condition itself, for both diabetes and HIV and in both cohorts: 2.57 against 1.43 for diabetes and 3.48 against 2.84 for HIV in the unfavourable outcome cohort, and 2.61 against 1.56 and 3.68 against 3.40 respectively for mortality. Second, non-recording of tobacco and alcohol status was concordant in 92.9% of records, which was leaning towards a form section left blank for various reasons that requires exploration to address the quality concerns. Third, restricting the analysis to records with a complete cases (i.e., where the data was available and recorded for behavioral and comorbidity records) left the estimates for the recorded categories unchanged to within 2.1%, so retaining the category did not distort the exposure estimates much. We therefore have to cautiously interpret the not-recorded coefficients as an indicator of incomplete programmatic assessment, and not as evidence about the underlying clinical or behavioural status of those patients.

One of the more nuanced findings was the role of weight as a predictive factor. Patients with low baseline body weight had significantly higher risks of unfavourable outcomes, supporting earlier studies highlighting malnutrition as a core determinant of TB mortality [20,23–25]. Approximately a quarter of the adult DSTB patients in Maharashtra had a pre-treatment body weight of less than 40 kg, comparable to other studies [26], making the findings generalizable to the overall population. Given the known association between undernutrition and TB outcomes in India, there is a compelling need to integrate and monitor regular anthropometric assessments and the findings have practical relevance in the context of the recently released National Guidance on Differentiated TB Care, which recommends risk stratification and severity assessment of all notified patients at diagnosis [27]. Substance use (tobacco or alcohol consumption) that was found to be associated with unfavourable outcomes could be a potential triaging variable. Patients identified with substance use could be flagged within Ni-kshay for enhanced adherence support, treatment monitoring, more frequent clinical review, and referral linkage to de-addiction services where available. This approach aligns with the Risk and Needs Assessment (RNA) tool piloted under the THALI project in Karnataka and Telangana, which identified substance use as a key predictor of poor outcomes [28].

Another observation from the study was the divergent relationship between EPTB and the two outcome measures. EPTB was associated with lower odds of unfavourable outcomes overall (aOR: 0.95, 95% CI: 0.92–0.98) but with slightly higher odds of mortality specifically (aOR: 1.10, 95% CI: 1.05–1.14). The slightly elevated mortality risk among EPTB patients likely reflects a combination of diagnostic delays inherent to the non-specific presentation of extrapulmonary disease, the involvement of high-fatality anatomical sites and differential age-groups getting affected with the disease. This finding is consistent with evidence from a large Chinese cohort in which age above 60 years increased the hazard of mortality by over 11-fold among patients with concurrent pulmonary and extrapulmonary disease, and by nearly 24-fold among those with exclusively EPTB [29]. Taken together, these observations support the case for including EPTB, particularly in older adults, as a criterion for early comprehensive clinical assessment and triaging within the differentiated care framework.

As an exploratory post-hoc analysis, we examined the time from treatment initiation to death in the mortality cohort using Kaplan-Meier estimates and Cox proportional hazards models, with follow-up censored at 365 days. This was carried out after the primary analysis was complete and was not part of the original analysis plan. The full description is given in supporting information S11 Table and S12 Table. The exploratory time-to-event post-hoc analysis pointed to two interesting findings. First, nearly two-thirds of the deaths occurred within the first two months of treatment, which suggests that measures intended to reduce TB deaths will need to act at diagnosis and during the intensive phase rather than across the whole regimen. The median time from treatment initiation to death was 38 days [IQR: 13–86], and 8,883 of the 14,261 deaths (62.3%) occurred within the first 2 months. The cumulative probability of death was 2.82% [2.76–2.88] at 2 months and 6.25% [6.04–6.47] at the end of year (Supporting information: S12 Table). Second, when follow-up was split at 2 months, the clinical risk factors changed little between the two periods (aHR for HIV: 3.10 [2.88–3.34] in the first 2 months; 3.11 [2.82–3.42] thereafter), whereas the “not recorded” or “unknown categories” reduced sharply (aHR for unrecorded HIV status: 4.91 [4.31–5.60] in the first 2 months; 1.12 [0.80–1.58] thereafter). These analyses were not pre-specified, and the estimates for the period after 2 months apply only to patients who survived the intensive phase and hence they are therefore presented as hypothesis-generating.

Despite its strengths, this study faced certain methodological challenges that have scope for improvement in future research. Future statistical modelling should expand beyond the two registers utilized here as well as datasets from recent years, as the programmatic database contains a wealth of additional patient-centric information. While the exclusion of those not initiated on treatment and those with missing baseline weights might have underestimated the risks, the sensitivity analyses performed through multiple imputation confirms the robustness of our primary findings. Missing data patterns (baseline body weight), while non-random, did not correlate strongly with outcomes, and multiple imputation yielded nearly consistent effect estimates comparable to the complete-case analysis. The same was observed when assessed using the identialcal and complete datasets for the clinical and behavioral risk factors (i.e., removing the records without any of these factors recorded). The consistency across analytical approaches strengthens the primary findings. Predictive mean matching, which ensures that imputed values match the observed data distribution, produced biologically plausible results without generating impossible values. In the analytic cohort, more than one in five patients had at least one of the four baseline assessments unrecorded, ranging from 0.7% for HIV status to 20.1% for alcohol intake. This makes the argument for improving the quality of data and designing the modules in Ni-kshay (e.g., the differentiate care module released recently) to help identify and separate a service gap from a patient refusal/non-offering or something that was offered and not captured.

Finally, the successful merging of notification and comorbidity registers using the “instance” variable in alignment with the latest programmatic norms and reporting framework could be a replicable method for future analyses. Majority of the studies that have utilized programmatic data in past have provided less clarity and utilized varying methodologies on which identifier was the identifier used for counting cases and subsequent analysis [22,30–41]. Hence, we also propose a standardized reporting framework (Table 3) that future researchers and program managers can adopt when conducting inferential analyses using TB-related programmatic data from Ni-kshay.

**Table 3:** Standardized reporting framework for studies utilizing Ni-kshay-based data.

Reporting framework elements	To detail the following
Data source	• Specify registers used (e.g., Notification Register, Comorbidity Register); • Filters applied to download the register and • Date or month and year of download.
Study population	• Inclusion/exclusion criteria (e.g., DSTB only, age ≥ 15 years); • Definition of denominators
Data cleaning	List of preprocessing steps followed including a flowchart: • duplicate removal, • variable/registers merging • handling of missing fields,
Variable definitions	Operational definitions for outcome categories (e.g., unfavourable outcomes), explanatory variables followed.
Analytical methods	• Describe statistical tests (e.g., chi-square, logistic regression), software used, adjustments (if-any). • Describe the variable used for counting as well as merging different registers from Ni-kshay (in this case, “instance”). • Describe the type of inferential analysis being carried (if applicable).
Results presentation	• Univariate and multivariate analysis outputs (e.g., Unadjusted and adjusted odds ratios with 95% CI); • clearly labelled forest plots and tables (as applicable)
Limitations	Coverage gaps, potential biases (e.g., non-response, misclassification), and system constraints
Ethical approval	• IRB details (if applicable) and • Administrative approval from the Central TB Division or State TB Office or the District TB Office

## Conclusion

This study offers a robust, replicable analytical framework grounded in demographic, clinical, and behavioural indicators for understanding the predictors of treatment outcomes using the programmatic data adhering to the updated norms, guidelines and definitions. Future work should expand this analysis to other Indian states and incorporate more recent data for longer timelines. Furthermore, investigating the impact of additional interventions available from the programmatic database, such as direct benefit transfer, treatment support systems and patient adherence levels using advanced statistical methods, can enable real-time risk stratification and better inform evidence-based public health interventions, as well as monitor the impact of the newer strategies on reducing the unfavourable outcomes and mortality.

## Supporting information

S1 Table Descriptive analysis of the adult, DSTB cohort for 2021 and 2022 with successful and unfavourable treatment outcomes. (DOCX)

S2 Table Descriptive analysis of the adult, DSTB cohort for 2021 and 2022 with successful treatment outcome and mortality. (DOCX)

S3 Table Adjusted odds ratios (aOR) of various risk factors against unfavourable treatment outcomes and mortality for 2021 and 2022. (DOCX)

S4 Table Age and weight-wise categorisation of different outcomes (successful outcomes, unfavourable outcomes and mortality) among cases across the two years combined. (DOCX)

S5 Table Pairwise Cramér’s V for all 28 pairs of the eight categorical predictors, both outcomes including after creating a composite substance use as a variable. (DOCX)

S6a Table Description of baseline characteristics between complete and missing weight data: unfavourable outcomes dataset (N  343,479). (DOCX)

S6b Table Description of baseline characteristics between complete and missing weight data: mortality dataset (N = 335,334). (DOCX)

S7 Table Adjusted odds ratios after multiple imputation analysis using predictive mean matching, for unfavourable outcomes and mortality. (DOCX)

S8 Table Variance inflation factors for the models fitted in the primary analysis, both outcomes. (DOCX)

S9a Table Adjusted odds ratios for an unfavourable treatment outcome under the missing-indicator specification (Model A) and restricted to records with a status recorded for all four variables (Model B). (DOCX)

S9b Table Adjusted odds ratios for death during treatment under the missing-indicator specification (Model A) and restricted to records with a status recorded for all four variables (Model B). (DOCX)

S10 Table Completeness of the baseline comorbidity and behavioural assessment, both outcomes. (DOCX)

S11 Table Adjusted hazard ratios for death during tuberculosis treatment over the whole follow-up period and within each period of the treatment course, mortality cohort. (DOCX)

S12 Table Cumulative probability of death at at 2 months from treatment initiation, mortality cohort. (DOCX)
