# Supplementary material for: Predictors of treatment outcomes in adults with drug–sensitive Tuberculosis in Maharashtra, India: A retrospective study
Source: PLOS Glob Public Health. 2026 Nov 6;6(11):e0006539. doi: 10.1371/journal.pgph.0006539 (PMC13743022; doi:10.1371/journal.pgph.0006539)
Supplement: Supplementary file 1 [file pgph.0006539.s001.docx]

**Supporting information**

**S1_Table. Descriptive analysis of the adult, DSTB cohort for 2021 and 2022 with successful and unfavourable treatment outcomes**

| Variable | 2021 | | | | 2022 | | | |
| --- | --- | --- | --- | --- | --- | --- | --- | --- |
|  | Successful treatment outcome  (n=128,520) (%) | Unfavourable treatment outcome  (n=10,124) (%) | Total  (n=138,644) | P-value | Successful treatment outcome  (n=172,798) (%) | Unfavourable treatment outcome  (n=11,682) (%) | Total  (n=184,480) | P-value |
| Age |  |  |  |  |  |  |  |  |
| 15-29 years | 46,444  (36.1) | 1785  (17.6) | 48,229  (34.8) | <0.001 | 58,000  (33.6) | 1907  (16.3) | 59,907  (32.5) | <0.001 |
| 30-44 years | 35,676  (27.7) | 2583  (25.5) | 38,259  (27.6) |  | 47,678  (27.6) | 2840  (24.3) | 50,518  (27.4) |  |
| 45-59 years | 26,087  (20.3) | 2660  (26.3) | 28,747  (20.7) |  | 35,999  (20.8) | 2989  (25.6) | 38,988  (21.1) |  |
| 60 years & above | 20,313  (15.8) | 3096  (30.6) | 23,409  (16.9) |  | 31,121  (18) | 3946  (33.8) | 35,067  (19) |  |
| Gender |  |  |  |  |  |  |  |  |
| Female | 60,138  (46.8) | 3604  (35.6) | 63,742  (46.9) | <0.001 | 78,686  (45.5) | 3910  (33.5) | 82,596  (44.8) | <0.001 |
| Male | 68,319  (53.2) | 6513  (64.3) | 74,832  (54) |  | 94,019  (54.4) | 7758  (66.4) | 101,777  (55.2) |  |
| Transgender | 63  (0.1) | 7  (0.1) | 70  (0.1) |  | 93  (0.1) | 14  (0.1) | 107  (0.1) |  |
| Tobacco consumption – Yes | 8197  (6.4) | 971  (9.6) | 9168  (6.6) | <0.001 | 10,769  (6.2) | 1055  (9) | 11,824  (6.4) | <0.001 |
| Tobacco consumption – Unknown | 23,364  (18.2) | 2213  (21.9) | 25,577  (18.5) |  | 30,452  (17.7) | 2690  (23.1) | 33,142  (18) |  |
| H/o alcohol consumption – Yes | 4477  (3.5) | 690  (6.8) | 5167  (3.7) | <0.001 | 5796  (3.4) | 754  (6.5) | 6550  (3.6) | <0.001 |
| H/o alcohol consumption – Unknown | 25,956  (20.2) | 2434  (24.1) | 28,390  (20.5) |  | 33,532  (19.4) | 2947  (25.2) | 36,479  (19.8) |  |
| Diabetes status - Yes | 7669  (6) | 1049  (10.4) | 8718  (6.3) | <0.001 | 10,196  (5.9) | 1132  (9.7) | 11,328  (6.1) | <0.001 |
| Diabetes status - unknown | 4070  (3.2) | 631  (6.2) | 4701  (3.4) |  | 2250  (1.3) | 469  (4) | 2719  (1.5) |  |
| HIV status – PLHIV | 3788  (3) | 876  (8.7) | 4664  (3.4) | <0.001 | 4428  (2.6) | 832  (7.1) | 5260  (2.9) | <0.001 |
| HIV status - Unknown | 995  (0.8) | 230  (2.3) | 1225  (0.9) |  | 727  (0.4) | 163  (1.4) | 890  (0.5) |  |
| Weight |  |  |  |  |  |  |  |  |
| >80 Kg | 1643  (1.3) | 105  (1.04) | 1748  (1.3) | <0.001 | 2484  (1.4) | 133  (1.1) | 2617  (1.4) | <0.001 |
| 61-80 Kg | 14,587  (11.4) | 958  (9.5) | 15,545  (11.2) |  | 19,962  (11.6) | 1094  (9.4) | 21,056  (11.4) |  |
| 41-60 Kg | 78,083  (60.8) | 5559  (54.9) | 83,642  (60.3) |  | 104,655  (60.6) | 6351  (54.4) | 111,006  (60.2) |  |
| 21-40 Kg | 33,920  (26.4) | 3457  (34.2) | 37,377  (27) |  | 45,299  (26.2) | 4054  (34.7) | 49,353  (26.8) |  |
| <20 Kg | 287  (0.2) | 45  (0.4) | 332  (0.2) |  | 398  (0.2) | 50  (0.4) | 448  (0.2) |  |
| Site: Extra-pulmonary | 46,953  (36.5) | 3039  (30.0) | 49,992  (36.1) | <0.001 | 62,626  (36.2) | 3350 (28.9) | 65,976  (35.8) | <0.001 |
