# Supplementary material for: Predictors of treatment outcomes in adults with drug–sensitive Tuberculosis in Maharashtra, India: A retrospective study
Source: PLOS Glob Public Health. 2026 Nov 6;6(11):e0006539. doi: 10.1371/journal.pgph.0006539 (PMC13743022; doi:10.1371/journal.pgph.0006539)
Supplement: Supplementary file 2 [file pgph.0006539.s002.docx]

**Supporting information**

**S2_Table. Descriptive analysis of the adult, DSTB cohort for 2021 and 2022 with successful treatment outcome and mortality**

| Variable | 2021 | | | | 2022 | | | |
| --- | --- | --- | --- | --- | --- | --- | --- | --- |
|  | Successful treatment outcome  (n=128,520) (%) | Died  (n=6473) (%) | Total  (n=134,993) | P-value | Successful treatment outcome  (n=172,798) (%) | Died  (n=7788)  (%) | Total  (n=180,586) | P-value |
| Age |  |  |  |  |  |  |  |  |
| 15-29 years | 46,444  (36.1) | 745  (11.5) | 47,189  (35) | <0.001 | 58,000  (33.6) | 759  (9.8) | 58,759  (32.5) | <0.001 |
| 30-44 years | 35,676  (27.8) | 1409  (21.8) | 37,085  (27.5) |  | 47,678  (27.6) | 1653  (21.2) | 49,331  (27.3) |  |
| 45-59 years | 26,087  (20.3) | 1802  (27.8) | 27,889  (20.7) |  | 35,999  (20.8) | 2102  (27) | 38,101  (21.1) |  |
| 60 years & above | 20,313  (15.8) | 2517  (38.9) | 22,830  (16.9) |  | 31,121  (18) | 3274  (42) | 34,395  (19.1) |  |
| Gender |  |  |  |  |  |  |  |  |
| Female | 60,138  (47.8) | 2228  (34.4) | 62,366  (46.2) | <0.001 | 78,686  (45.5) | 2580  (33.1) | 81,266  (45) | <0.001 |
| Male | 68,319  (53.2) | 4240  (65.5) | 72,559  (53.7) |  | 94,019  (54.4) | 5199  (66.8) | 99,218  (54.9) |  |
| Transgender | 63  (0.1) | 5  (0.1) | 68  (0.1) |  | 93  (0.1) | 9  (0.1) | 102  (0.1) |  |
| Tobacco consumption – Yes | 8197  (6.4) | 602  (9.3) | 8799  (6.5) | <0.001 | 10,769  (6.2) | 706  (9.1) | 11,475  (6.4) | <0.001 |
| Tobacco consumption – Unknown | 23,364  (18.2) | 1394  (21.6) | 24,758  (18.4) |  | 30,452  (17.7) | 1819  (23.4) | 32,271  (17.9) |  |
| H/o alcohol consumption – Yes | 4477  (3.5) | 422  (6.5) | 4899  (3.6) | <0.001 | 5796  (3.4) | 492  (6.3) | 6288  (3.5) | <0.001 |
| H/o alcohol consumption – Unknown | 25,956  (20.2) | 1514  (23.4) | 27,470  (20.4) |  | 33,532  (19.4) | 2006  (25.8) | 35,538  (19.7) |  |
| Diabetes status - Yes | 7669  (6) | 768  (11.9) | 8437  (6.3) | <0.001 | 10,196  (5.9) | 892  (11.5) | 11,088  (6.1) | <0.001 |
| Diabetes status - unknown | 4070  (3.2) | 398  (6.2) | 4468  (3.3) |  | 2250  (1.3) | 322  (4.1) | 2572  (1.4) |  |
| HIV status – PLHIV | 3788  (3) | 666  (10.3) | 4454  (3.3) | <0.001 | 4428  (2.6) | 619  (8) | 5047  (2.8) | <0.001 |
| HIV status - Unknown | 995  (0.8) | 158  (2.4) | 1153  (0.9) |  | 727  (0.4) | 106  (1.4) | 833  (0.5) |  |
| Weight |  |  |  |  |  |  |  |  |
| >80 Kg | 1643  (1.3) | 59  (0.9) | 1702  (1.3) | <0.001 | 2484  (1.4) | 75  (0.9) | 2559  (1.4) | <0.001 |
| 61-80 Kg | 14,587  (11.4) | 575  (8.9) | 15,162  (11.2) |  | 19,962  (11.6) | 648  (8.3) | 20,610  (11.4) |  |
| 41-60 Kg | 78,083  (60.8) | 3429  (53) | 81,512  (60.4) |  | 104,655  (60.6) | 4068  (52.2) | 108,723  (60.2) |  |
| 21-40 Kg | 33,920  (26.4) | 2373  (36.7) | 36,293  (26.9) |  | 45,299  (26.2) | 2952  (37.9) | 48,251  (26.7) |  |
| <20 Kg | 287  (0.2) | 37  (0.6) | 324  (0.2) |  | 398  (0.2) | 45  (0.6) | 443  (0.2) |  |
| Site: Extra-pulmonary | 46,953  (36.5) | 2057  (31.8) | 49,010  (36.3) | <0.001 | 62,626  (36.2) | 2242  (28.8) | 64,868  (35.9) | <0.001 |
