# Supplementary material for: Predictors of treatment outcomes in adults with drug–sensitive Tuberculosis in Maharashtra, India: A retrospective study
Source: PLOS Glob Public Health. 2026 Nov 6;6(11):e0006539. doi: 10.1371/journal.pgph.0006539 (PMC13743022; doi:10.1371/journal.pgph.0006539)
Supplement: Supplementary file 3 [file pgph.0006539.s003.docx]

**Supporting information**

**S3_Table. Adjusted* odds ratios (aOR) of various risk factors against unfavourable treatment outcomes and mortality for 2021 and 2022**

| Factor | Unfavourable outcomes | | | | Mortality | | | |
| --- | --- | --- | --- | --- | --- | --- | --- | --- |
|  | 2021 | | 2022 | | 2021 | | 2022 | |
|  | aOR | 95% CI | aOR | 95% CI | aOR | 95% CI | aOR | 95% CI |
| Age |  |  |  |  |  |  |  |  |
| 15-29 years | Ref | - | Ref | - | Ref | - | Ref | - |
| 30-44 years | 1.95 | 1.83-2.08 | 1.86 | 1.75-1.98 | 2.63 | 2.40-2.88 | 2.83 | 2.59-3.09 |
| 45-59 years | 2.74 | 2.58-2.92 | 2.57 | 2.42-2.73 | 4.63 | 4.24-5.06 | 4.76 | 4.37-5.18 |
| 60 years & above | 4.01 | 3.76-4.26 | 3.79 | 3.57-4.01 | 8.08 | 7.42-8.80 | 8.20 | 7.56-8.89 |
| Gender - Female | Ref | - | Ref | - | Ref | - | Ref | - |
| Gender – Male | 1.45 | 1.38-1.51 | 1.55 | 1.49-1.62 | 1.44 | 1.36-1.52 | 1.49 | 1.42-1.57 |
| Gender – Transgender | 1.85 | 0.84-4.09 | 3.11 | 1.76-5.49 | 2.14 | 0.84-5.41 | 3.05 | 1.52-6.15 |
| Tobacco consumption - No | Ref | - | Ref | - | Ref | - | Ref | - |
| Tobacco consumption – Yes | 1.20 | 1.12-1.30 | 1.19 | 1.11-1.27 | 1.08 | 0.99-1.18 | 1.12 | 1.03-1.22 |
| Tobacco consumption – Unknown | 1.36 | 1.29-1.43 | 1.48 | 1.42-1.55 | 1.35 | 1.27-1.44 | 1.53 | 1.45-1.62 |
| H/o alcohol consumption - No | Ref | - | Ref | - | Ref | - | Ref | - |
| H/o alcohol consumption – Yes | 1.66 | 1.52-1.81 | 1.67 | 1.54-1.81 | 1.52 | 1.36-1.69 | 1.60 | 1.45-1.77 |
| H/o alcohol consumption – Unknown | 1.36 | 1.30-1.43 | 1.51 | 1.44-1.57 | 1.33 | 1.25-1.41 | 1.57 | 1.49-1.66 |
| Diabetes - No | Ref | - | Ref | - | Ref | - | Ref | - |
| Diabetes status - Yes | 1.45 | 1.35-1.55 | 1.40 | 1.31-1.50 | 1.54 | 1.42-1.67 | 1.57 | 1.46-1.70 |
| Diabetes status - unknown | 2.08 | 1.90-2.27 | 3.28 | 2.96-3.64 | 2.06 | 1.85-2.30 | 3.47 | 3.06-3.92 |
| HIV status - negative | Ref | - | Ref | - | Ref | - | Ref | - |
| HIV status – PLHIV | 2.90 | 2.68-3.14 | 2.74 | 2.54-2.97 | 3.67 | 3.35-4.01 | 3.14 | 2.87-3.43 |
| HIV status - Unknown | 3.21 | 2.76-3.72 | 3.65 | 3.07-4.35 | 3.55 | 2.97-4.24 | 3.68 | 2.97-4.55 |
| Weight |  |  |  |  |  |  |  |  |
| >80 Kg | 1.04 | 0.84-1.28 | 1.02 | 0.85-1.23 | 1.01 | 0.76-1.32 | 1.00 | 0.79-1.28 |
| 61-80 Kg | Ref | - | Ref | - | Ref | - | Ref | - |
| 41-60 Kg | 1.22 | 1.13-1.31 | 1.21 | 1.13-1.29 | 1.29 | 1.18-1.41 | 1.34 | 1.23-1.46 |
| 21-40 Kg | 2.14 | 1.99-2.31 | 2.14 | 2.00-2.30 | 2.66 | 2.42-2.92 | 2.80 | 2.56-3.06 |
| <20 Kg | 3.58 | 2.57-4.97 | 3.10 | 2.29-4.21 | 5.61 | 3.89-8.11 | 5.21 | 3.75-7.24 |
| Site Pulmonary | Ref | - | Ref | - | Ref | - | Ref | - |
| Site: Extra-pulmonary | 0.97 | 0.92-1.01 | 0.93 | 0.89-0.97 | 1.17 | 1.10-1.24 | 1.04 | 0.98-1.09 |

**Adjusted for age, gender and weight in the multivariate regression; CI – confidence interval*
