# Supplementary material for: Predictors of treatment outcomes in adults with drug–sensitive Tuberculosis in Maharashtra, India: A retrospective study
Source: PLOS Glob Public Health. 2026 Nov 6;6(11):e0006539. doi: 10.1371/journal.pgph.0006539 (PMC13743022; doi:10.1371/journal.pgph.0006539)
Supplement: Supplementary file 4 [file pgph.0006539.s004.docx]

**Supporting information**

**S4_Table. Age and weight-wise categorisation of different outcomes (successful outcomes, unfavourable outcomes and mortality) among cases across the 2 years combined**

| Successful outcomes (n=301,318) (%) | | | | | | |
| --- | --- | --- | --- | --- | --- | --- |
| Age | Weight >80Kg | Weight: 61-80Kg | Weight: 41-60Kg | Weight: 21-40Kg | Weight: <20Kg | Total |
| 15-29 years | 923 (0.9) | 7020 (6.7) | 59,026 (56.5) | 37,111 (35.5) | 364 (0.4) | 1,04,444 |
| 30-44 years | 1533 (1.8) | 11,497 (13.8) | 51,889 (62.3) | 18,293 (21.9) | 142 (0.2) | 83,354 |
| 45-59 years | 1072 (1.7) | 9385 (15.1) | 39,624 (63.8) | 11,905 (19.2) | 100 (0.2) | 62,086 |
| 60 & above years | 599 (1.2) | 6647 (12.9) | 32,199 (62.6) | 11,910 (23.2) | 79 (0.2) | 51,434 |
| Total | 4127 (1.4) | 34,549 (11.5) | 182,738 (60.6) | 79,219 (26.3) | 685 (0.2) | 3,01,318 |
| Unfavourable outcomes (n=21,806) (%) | | | | | | |
| Age | Weight >80Kg | Weight: 61-80Kg | Weight: 41-60Kg | Weight: 21-40Kg | Weight: <20Kg | Total |
| 15-29 years | 27 (0.7) | 173 (4.7) | 1759 (47.6) | 1705 (46.2) | 28 (0.8) | 3692 |
| 30-44 years | 62 (1.1) | 402 (7.4) | 2889 (53.3) | 2053 (37.9) | 17 (0.3) | 5423 |
| 45-59 years | 66 (1.2) | 615 (10.9) | 3220 (57) | 1728 (30.6) | 20 (0.4) | 5649 |
| 60 & above years | 83 (1.2) | 862 (12.2) | 4042 (57.4) | 2025 (28.8) | 30 (0.4) | 7042 |
| Total | 238 (1.1) | 2052 (9.4) | 11,910 (54.6) | 7511 (34.4) | 95 (0.4) | 21,806 |
| Mortality (n=14,261) (%) | | | | | | |
| Age | Weight >80Kg | Weight: 61-80Kg | Weight: 41-60Kg | Weight: 21-40Kg | Weight: <20Kg | Total |
| 15-29 years | 8 (0.5) | 39 (2.6) | 550 (36.6) | 886 (58.9) | 21 (1.4) | 1504 |
| 30-44 years | 21 (0.7) | 159 (5.2) | 1485 (48.5) | 1384 (45.2) | 13 (0.4) | 3062 |
| 45-59 years | 42 (1.1) | 360 (9.2) | 2151 (55.1) | 1331 (34.1) | 20 (0.5) | 3904 |
| 60 & above years | 63 (1.1) | 665 (11.5) | 3311 (57.2) | 1724 (29.8) | 28 (0.5) | 5791 |
| Total | 134 (0.9) | 1223 (8.6) | 7497 (52.6) | 5325 (37.3) | 82 (0.6) | 14,261 |
