# Supplementary material for: Predictors of treatment outcomes in adults with drug–sensitive Tuberculosis in Maharashtra, India: A retrospective study
Source: PLOS Glob Public Health. 2026 Nov 6;6(11):e0006539. doi: 10.1371/journal.pgph.0006539 (PMC13743022; doi:10.1371/journal.pgph.0006539)
Supplement: Supplementary file 5 [file pgph.0006539.s005.docx]

**Supporting information**

**S5_Table. Pairwise Cramér's V for all 28 pairs of the eight categorical predictors, both outcomes**

| **Pair of variables** | **Cramér's V, unfavourable** | **Cramér's V, mortality** |
| --- | --- | --- |
| current tobacco use × history of alcohol intake | 0.713 | 0.712 |
| diabetes status × HIV status | 0.314 | 0.312 |
| diabetes status × current tobacco use | 0.219 | 0.217 |
| diabetes status × history of alcohol intake | 0.206 | 0.204 |
| pre-treatment weight × site of disease | 0.186 | 0.185 |
| age group × diabetes status | 0.147 | 0.147 |
| age group × gender | 0.144 | 0.145 |
| gender × site of disease | 0.138 | 0.137 |
| gender × current tobacco use | 0.135 | 0.134 |
| gender × pre-treatment weight | 0.132 | 0.134 |
| age group × site of disease | 0.120 | 0.122 |
| gender × history of alcohol intake | 0.118 | 0.117 |
| HIV status × current tobacco use | 0.107 | 0.106 |
| HIV status × history of alcohol intake | 0.101 | 0.099 |
| age group × pre-treatment weight | 0.098 | 0.099 |
| pre-treatment weight × diabetes status | 0.083 | 0.083 |
| current tobacco use × site of disease | 0.081 | 0.081 |
| age group × current tobacco use | 0.077 | 0.077 |
| age group × HIV status | 0.076 | 0.076 |
| history of alcohol intake × site of disease | 0.068 | 0.067 |
| diabetes status × site of disease | 0.067 | 0.067 |
| age group × history of alcohol intake | 0.064 | 0.064 |
| gender × diabetes status | 0.043 | 0.044 |
| pre-treatment weight × history of alcohol intake | 0.032 | 0.032 |
| pre-treatment weight × current tobacco use | 0.031 | 0.031 |
| gender × HIV status | 0.026 | 0.025 |
| pre-treatment weight × HIV status | 0.022 | 0.022 |
| HIV status × site of disease | 0.010 | 0.010 |
| age * Substance use* | 0.074 | 0.073 |
| Pre-treatment weight x Substance use* | 0.024 | 0.024 |
| diabetes status x, Substance use | 0.203 | 0.201 |
| substance use x site of disease* | 0.088 | 0.088 |

All 28 pairs are shown, ordered by the value in the unfavourable outcome cohort. ***** Additional 4 pairs with substance use as a composite variable created and checked against is reported here
