# Supplementary material for: Predictors of treatment outcomes in adults with drug–sensitive Tuberculosis in Maharashtra, India: A retrospective study
Source: PLOS Glob Public Health. 2026 Nov 6;6(11):e0006539. doi: 10.1371/journal.pgph.0006539 (PMC13743022; doi:10.1371/journal.pgph.0006539)
Supplement: Supplementary file 6 [file pgph.0006539.s006.docx]

**Supporting information**

**S6a_Table. Description of baseline characteristics between complete and missing weight data: Unfavourable outcomes dataset (N = 343,479)**

| Characteristic | Patients with complete data (n=323,124) | Patients with missing weight (n=20,355) |
| --- | --- | --- |
| Age, mean (SD) | 40.6 (17.2) | 40.1 (16.9) |
| Age group, n (%) |  |  |
| 15-29 | 108,136 (33.5) | 6,850 (33.7) |
| 30-44 | 88,777 (27.5) | 5,819 (28.6) |
| 45-59 | 67,735 (21.0) | 4,306 (21.2) |
| ≥60 | 58,476 (18.1) | 3,380 (16.6) |
| Gender, n (%) |  |  |
| Female | 146,338 (45.3) | 9,021 (44.3) |
| Male | 176,609 (54.7) | 11,324 (55.6) |
| Transgender | 177 (0.1) | 10 (0.0) |
| Diabetes, n (%) |  |  |
| No | 295,658 (91.5) | 18,384 (90.3) |
| Yes | 20,046 (6.2) | 1,392 (6.8) |
| Unknown | 7,420 (2.3) | 579 (2.8) |
| HIV status, n (%) |  |  |
| Negative | 311,085 (96.3) | 19,524 (95.9) |
| Positive | 9,924 (3.1) | 681 (3.3) |
| Unknown | 2,115 (0.7) | 150 (0.7) |
| Substance use, n (%) |  |  |
| None | 231,326 (71.6) | 14,558 (71.5) |
| Tobacco only | 12,400 (3.8) | 918 (4.5) |
| Alcohol only | 3,125 (1.0) | 298 (1.5) |
| Both | 8,592 (2.7) | 682 (3.4) |
| Unknown | 67,681 (20.9) | 3,899 (19.2) |
| TB site, n (%) |  |  |
| Pulmonary | 207,156 (64.1) | 13,765 (67.6) |
| Extrapulmonary | 115,968 (35.9) | 6,590 (32.4) |
| Treatment outcome: Unfavourable | 21,806 (6.8) | 1,459 (7.2) |
