# Supplementary material for: Predictors of treatment outcomes in adults with drug–sensitive Tuberculosis in Maharashtra, India: A retrospective study
Source: PLOS Glob Public Health. 2026 Nov 6;6(11):e0006539. doi: 10.1371/journal.pgph.0006539 (PMC13743022; doi:10.1371/journal.pgph.0006539)
Supplement: Supplementary file 7 [file pgph.0006539.s007.docx]

**Supporting information**

**S6b_Table. Description of baseline characteristics between complete and missing weight data: Mortality dataset (N = 335,334)**

| Characteristic | Patients with Complete data (n=315,579) | Patients with Missing weight (n=19,755) |
| --- | --- | --- |
| Age, mean (SD) | 40.6 (17.3) | 40.1 (16.9) |
| Age group, n (%) |  |  |
| 15-29 | 105,948 (33.6) | 6,690 (33.9) |
| 30-44 | 86,416 (27.4) | 5,611 (28.4) |
| 45-59 | 65,990 (20.9) | 4,168 (21.1) |
| ≥60 | 57,225 (18.1) | 3,286 (16.6) |
| Gender, n (%) |  |  |
| Female | 143,632 (45.5) | 8,829 (44.7) |
| Male | 171,777 (54.4) | 10,916 (55.3) |
| Transgender | 170 (0.1) | 10 (0.1) |
| Diabetes, n (%) |  |  |
| No | 289,014 (91.6) | 17,876 (90.5) |
| Yes | 19,525 (6.2) | 1,349 (6.8) |
| Unknown | 7,040 (2.2) | 530 (2.7) |
| HIV status, n (%) |  |  |
| Negative | 304,092 (96.4) | 18,972 (96.0) |
| Positive | 9,501 (3.0) | 655 (3.3) |
| Unknown | 1,986 (0.6) | 128 (0.6) |
| Substance use, n (%) |  |  |
| None | 226,571 (71.8) | 14,194 (71.9) |
| Tobacco only | 12,048 (3.8) | 882 (4.5) |
| Alcohol only | 2,961 (0.9) | 278 (1.4) |
| Both | 8,226 (2.6) | 651 (3.3) |
| Unknown | 65,773 (20.8) | 3,750 (19.0) |
| TB site, n (%) |  |  |
| Pulmonary | 201,701 (63.9) | 13,350 (67.6) |
| Extrapulmonary | 113,878 (36.1) | 6,405 (32.4) |
| Treatment outcome: Mortality, n (%) | 14,261 (4.5) | 859 (4.3) |
