# Supplementary material for: Predictors of treatment outcomes in adults with drug–sensitive Tuberculosis in Maharashtra, India: A retrospective study
Source: PLOS Glob Public Health. 2026 Nov 6;6(11):e0006539. doi: 10.1371/journal.pgph.0006539 (PMC13743022; doi:10.1371/journal.pgph.0006539)
Supplement: Supplementary file 8 [file pgph.0006539.s008.docx]

**Supporting information**

**S7_Table. Adjusted odds ratios after multiple imputation analysis using “Predictive Mean Matching (PMM)” for unfavourable outcomes and mortality**

| Variable | Category | Unfavourable: PMM aOR (95% CI) | Mortality: PMM aOR (95% CI) |
| --- | --- | --- | --- |
| Weight | >80 kg | 1.04 (0.90-1.19) | 1.00 (0.97-1.21) |
|  | 61-80 kg (ref) | 1.00 | 1.00 |
|  | 41-60 kg | 1.19 (1.13-1.25) | 1.32 (1.25-1.42) |
|  | 21-40 kg | 2.01 (1.90-2.12) | 2.70 (2.53-2.89) |
|  | ≤20 kg | 3.10 (2.48-3.89) | 5.17 (4.04-6.61) |
| Age | 15-29 (ref) | 1.00 | 1.00 |
|  | 30-44 | 1.74 (1.66-1.81) | 2.45 (2.30-2.60) |
|  | 45-59 | 2.31 (2.21-2.41) | 4.03 (3.79-4.28) |
|  | ≥60 | 3.63 (3.49-3.79) | 7.66 (7.23-8.12) |
| Gender | Female (ref) | 1.00 | 1.00 |
|  | Male | 1.42 (1.38-1.46) | 1.43 (1.37-1.48) |
| Diabetes | No (ref) | 1.00 | 1.00 |
|  | Yes | 1.43 (1.36-1.49) | 1.59 (1.50-1.68) |
|  | Unknown | 1.79 (1.66-1.94) | 1.72 (1.56-1.90) |
| HIV | Negative (ref) | 1.00 | 1.00 |
|  | Positive | 2.79 (2.65-2.95) | 3.35 (3.15-3.57) |
|  | Unknown | 1.95 (1.72-2.21) | 2.08 (1.78-2.42) |
| Substance | None (ref) | 1.00 | 1.00 |
|  | Tobacco only | 0.99 (0.92-1.05) | 0.89 (0.82-0.97) |
|  | Alcohol only | 1.93 (1.74-2.13) | 1.77 (1.56-2.02) |
|  | Both | 1.54 (1.44-1.65) | 1.46 (1.34-1.59) |
|  | Unknown | 1.27 (1.23-1.31) | 1.28 (1.23-1.33) |
| TB site | Pulmonary (ref) | 1.00 | 1.00 |
|  | Extrapulmonary | 0.95 (0.92-0.98) | 1.08 (1.04-1.12) |
