# Supplementary material for: Predictors of treatment outcomes in adults with drug–sensitive Tuberculosis in Maharashtra, India: A retrospective study
Source: PLOS Glob Public Health. 2026 Nov 6;6(11):e0006539. doi: 10.1371/journal.pgph.0006539 (PMC13743022; doi:10.1371/journal.pgph.0006539)
Supplement: Supplementary file 9 [file pgph.0006539.s009.docx]

**Supporting information**

**S8_Table. Variance inflation factors (VIF) for the models fitted in the primary analysis, both outcomes**

| **Model** | **Largest VIF, unfavourable** | **Indicator carrying it** | **Largest VIF, mortality** |
| --- | --- | --- | --- |
| Age group, gender and weight | 2.560 | pre-treatment weight: 21-40 kg | 2.559 |
| and diabetes status | 2.580 | pre-treatment weight: 21-40 kg | 2.579 |
| and HIV status | 2.564 | pre-treatment weight: 21-40 kg | 2.563 |
| and current tobacco use | 2.572 | pre-treatment weight: 21-40 kg | 2.570 |
| and history of alcohol intake | 2.571 | pre-treatment weight: 21-40 kg | 2.570 |
| and site of disease | 2.675 | pre-treatment weight: 21-40 kg | 2.673 |
| Composite substance-use specification | 2.719 | pre-treatment weight: 21-40 kg | 2.717 |
| All eight covariates (diagnostic purpose only)* | 3.959 | current tobacco use: not recorded | 3.956 |

In the primary analysis each exposure is entered into a separate model alongside age group, gender and pre-treatment weight, so current tobacco use and history of alcohol intake never appear together. (*)The final row is reported as a collinearity diagnostic on the design matrix only; no effect estimates from a model containing all eight covariates simultaneously are reported anywhere in this paper. Values above 5 were taken to warrant attention; no value in any model reached 5. The association detected by the Cramér's V could potentially be an artefact of concordant non-recording. But the composite substance use variable was developed and used in the sensitivity analysis
