# Supplementary material for: Predictors of treatment outcomes in adults with drug–sensitive Tuberculosis in Maharashtra, India: A retrospective study
Source: PLOS Glob Public Health. 2026 Nov 6;6(11):e0006539. doi: 10.1371/journal.pgph.0006539 (PMC13743022; doi:10.1371/journal.pgph.0006539)
Supplement: Supplementary file 10 [file pgph.0006539.s010.docx]

**Supporting information**

**S9a_Table. Adjusted odds ratios for an unfavourable treatment outcome under the missing-indicator specification (Model A) and restricted to records with a status recorded for all four variables (Model B)**

| **Variable and category** | **Model A aOR** | **Model A 95% CI** | **Model B aOR** | **Model B 95% CI** | **Ratio B / A** |
| --- | --- | --- | --- | --- | --- |
| Diabetes status: Non-diabetic (reference) | 1.00 | Reference | 1.00 | Reference | 1.00 |
| Diabetes status: Diabetic | 1.43 | 1.36 to 1.50 | 1.44 | 1.36 to 1.52 | 1.00 |
| Diabetes status: Not recorded | 2.57 | 2.40 to 2.74 | — | — | — |
| HIV status: HIV negative (reference) | 1.00 | Reference | 1.00 | Reference | 1.00 |
| HIV status: Living with HIV | 2.84 | 2.69 to 3.00 | 2.84 | 2.66 to 3.03 | 1.00 |
| HIV status: Not recorded | 3.48 | 3.10 to 3.89 | — | — | — |
| Current tobacco use: No current tobacco use (reference) | 1.00 | Reference | 1.00 | Reference | 1.00 |
| Current tobacco use: Yes | 1.20 | 1.14 to 1.26 | 1.22 | 1.16 to 1.29 | 1.02 |
| Current tobacco use: Not recorded | 1.43 | 1.38 to 1.47 | — | — | — |
| History of alcohol intake: No history of alcohol intake (reference) | 1.00 | Reference | 1.00 | Reference | 1.00 |
| History of alcohol intake: Yes | 1.67 | 1.57 to 1.77 | 1.67 | 1.57 to 1.77 | 1.00 |
| History of alcohol intake: Not recorded | 1.44 | 1.39 to 1.49 | — | — | — |
| Site of disease: Pulmonary (reference) | 1.00 | Reference | 1.00 | Reference | 1.00 |
| Site of disease: Extra-pulmonary | 0.95 | 0.92 to 0.98 | 0.96 | 0.92 to 1.00 | 1.01 |

Both models use the specification of the primary analysis: each exposure entered alongside age group, gender and pre-treatment weight. Model A retains records with no entry as a separate "not recorded" or “unknown” category and uses the whole cohort (n = 323,124). Model B is restricted to the 253,575 records with a status recorded for diabetes, HIV, tobacco and alcohol. The not-recorded category does not exist in Model B by construction, so no ratio is shown for those rows.
