# Supplementary material for: Predictors of treatment outcomes in adults with drug–sensitive Tuberculosis in Maharashtra, India: A retrospective study
Source: PLOS Glob Public Health. 2026 Nov 6;6(11):e0006539. doi: 10.1371/journal.pgph.0006539 (PMC13743022; doi:10.1371/journal.pgph.0006539)
Supplement: Supplementary file 11 [file pgph.0006539.s011.docx]

**Supporting information**

**S9b_Table. Adjusted odds ratios for death during treatment under the missing-indicator specification (Model A) and restricted to records with a status recorded for all four variables (Model B)**

| **Variable and category** | **Model A aOR** | **Model A 95% CI** | **Model B aOR** | **Model B 95% CI** | **Ratio B / A** |
| --- | --- | --- | --- | --- | --- |
| Diabetes status: Non-diabetic (reference) | 1.00 | Reference | 1.00 | Reference | 1.00 |
| Diabetes status: Diabetic | 1.56 | 1.48 to 1.65 | 1.56 | 1.46 to 1.66 | 1.00 |
| Diabetes status: Not recorded | 2.61 | 2.41 to 2.84 | — | — | — |
| HIV status: HIV negative (reference) | 1.00 | Reference | 1.00 | Reference | 1.00 |
| HIV status: Living with HIV | 3.40 | 3.19 to 3.63 | 3.39 | 3.14 to 3.65 | 1.00 |
| HIV status: Not recorded | 3.68 | 3.22 to 4.22 | — | — | — |
| Current tobacco use: No current tobacco use (reference) | 1.00 | Reference | 1.00 | Reference | 1.00 |
| Current tobacco use: Yes | 1.11 | 1.04 to 1.18 | 1.13 | 1.06 to 1.20 | 1.02 |
| Current tobacco use: Not recorded | 1.45 | 1.39 to 1.51 | — | — | — |
| History of alcohol intake: No history of alcohol intake (reference) | 1.00 | Reference | 1.00 | Reference | 1.00 |
| History of alcohol intake: Yes | 1.57 | 1.46 to 1.69 | 1.55 | 1.44 to 1.68 | 0.99 |
| History of alcohol intake: Not recorded | 1.46 | 1.40 to 1.52 | — | — | — |
| Site of disease: Pulmonary (reference) | 1.00 | Reference | 1.00 | Reference | 1.00 |
| Site of disease: Extra-pulmonary | 1.10 | 1.05 to 1.14 | 1.11 | 1.06 to 1.16 | 1.02 |

Model A, n = 315,579; Model B, n = 248,027.
