# Supplementary material for: Predictors of treatment outcomes in adults with drug–sensitive Tuberculosis in Maharashtra, India: A retrospective study
Source: PLOS Glob Public Health. 2026 Nov 6;6(11):e0006539. doi: 10.1371/journal.pgph.0006539 (PMC13743022; doi:10.1371/journal.pgph.0006539)
Supplement: Supplementary file 12 [file pgph.0006539.s012.docx]

**Supporting information**

**S10_Table. Completeness of the baseline comorbidity and behavioural assessment, both outcomes**

|  | **Unfavourable outcome cohort** | **Mortality cohort** |
| --- | --- | --- |
| Total records analysed | 323,124 | 315,579 |
| Records with all four statuses recorded | 253,575 | 248,027 |
| Records with at least one status not recorded | 69,549 | 67,552 |
| Diabetes status not recorded | 7,420 | 7,040 |
| HIV status not recorded | 2,115 | 1,986 |
| Current tobacco use not recorded | 59,192 | 57,493 |
| History of alcohol intake not recorded | 64,869 | 63,008 |
| Not recorded for both tobacco and alcohol | 54,989 | 53,401 |

Non-recording or unknown status is almost entirely confined to the two behavioural variables. It is also highly concordant between them: 92.9% of records with no tobacco entry also have no alcohol entry.
