# Supplementary material for: Predictors of treatment outcomes in adults with drug–sensitive Tuberculosis in Maharashtra, India: A retrospective study
Source: PLOS Glob Public Health. 2026 Nov 6;6(11):e0006539. doi: 10.1371/journal.pgph.0006539 (PMC13743022; doi:10.1371/journal.pgph.0006539)
Supplement: Supplementary file 13 [file pgph.0006539.s013.docx]

**Supporting information**

**S11_Table. Adjusted hazard ratios for mortality during tuberculosis treatment over the whole follow-up period and within each period of the treatment course, mortality cohort**

| **Variable and category** | **Whole follow-up, aHR (95% CI)** | **First 2 months, aHR (95% CI)** | **After 2 months, aHR (95% CI)** |
| --- | --- | --- | --- |
| **Age group** |  |  |  |
| **15 to 29 years (reference)** | **1.00** | **1.00** | **1.00** |
| 30 to 44 years | 2.73 (2.57 to 2.91) | 2.79 (2.57 to 3.02) | 2.64 (2.39 to 2.92) |
| 45 to 59 years | 4.62 (4.35 to 4.91) | 4.71 (4.36 to 5.09) | 4.48 (4.06 to 4.93) |
| 60 years and above | 7.82 (7.38 to 8.28) | 7.76 (7.21 to 8.36) | 7.91 (7.21 to 8.67) |
| **Gender** |  |  |  |
| **Female (reference)** | **1.00** | **1.00** | **1.00** |
| Male | 1.47 (1.42 to 1.52) | 1.43 (1.37 to 1.50) | 1.54 (1.45 to 1.63) |
| Transgender | 2.37 (1.40 to 4.00) | 1.95 (0.93 to 4.08) | 3.01 (1.43 to 6.33) |
| **Pre-treatment body weight** |  |  |  |
| Greater than 80 kg | 0.98 (0.82 to 1.17) | 1.06 (0.84 to 1.32) | 0.88 (0.65 to 1.17) |
| **61 to 80 kg (reference)** | **1.00** | **1.00** | **1.00** |
| 41 to 60 kg | 1.34 (1.26 to 1.42) | 1.34 (1.24 to 1.45) | 1.33 (1.21 to 1.47) |
| 21 to 40 kg | 2.74 (2.57 to 2.92) | 2.77 (2.55 to 3.00) | 2.70 (2.44 to 2.98) |
| 20 kg or less | 4.88 (3.90 to 6.10) | 5.06 (3.82 to 6.71) | 4.59 (3.18 to 6.64) |
| **Diabetes status** |  |  |  |
| **Non-diabetic (reference)** | **1.00** | **1.00** | **1.00** |
| Diabetic | 1.50 (1.42 to 1.58) | 1.53 (1.43 to 1.63) | 1.46 (1.34 to 1.59) |
| Not recorded | 2.53 (2.35 to 2.73) | 3.22 (2.95 to 3.50) | 1.40 (1.19 to 1.65) |
| **HIV status** |  |  |  |
| **HIV negative (reference)** | **1.00** | **1.00** | **1.00** |
| Living with HIV | 3.10 (2.93 to 3.29) | 3.10 (2.88 to 3.34) | 3.11 (2.82 to 3.42) |
| Not recorded | 3.45 (3.06 to 3.90) | 4.91 (4.31 to 5.60) | 1.12 (0.80 to 1.58) |
| **Current tobacco use** |  |  |  |
| **No current tobacco use (reference)** | **1.00** | **1.00** | **1.00** |
| Yes | 1.11 (1.05 to 1.18) | 1.11 (1.03 to 1.20) | 1.11 (1.01 to 1.22) |
| Not recorded | 1.39 (1.34 to 1.45) | 1.52 (1.45 to 1.60) | 1.20 (1.12 to 1.28) |
| **History of alcohol intake** |  |  |  |
| **No history of alcohol intake (reference)** | **1.00** | **1.00** | **1.00** |
| Yes | 1.53 (1.43 to 1.64) | 1.47 (1.35 to 1.61) | 1.65 (1.47 to 1.84) |
| Not recorded | 1.41 (1.35 to 1.46) | 1.52 (1.45 to 1.60) | 1.23 (1.15 to 1.31) |
| **Site of disease** |  |  |  |
| **Pulmonary (reference)** | **1.00** | **1.00** | **1.00** |
| Extra-pulmonary | 1.06 (1.02 to 1.10) | 1.09 (1.04 to 1.15) | 1.02 (0.96 to 1.08) |
| Observations | 315,579 | 315,579 | 306,258 |

aHR, adjusted hazard ratio; CI, confidence interval. Cox proportional hazards models using the specification of the primary analysis: age group, gender and pre-treatment weight entered together, and each of diabetes status, HIV status, current tobacco use, history of alcohol intake and site of disease entered into a separate model containing that variable together with age group, gender and pre-treatment weight. Follow-up runs from treatment initiation and is censored administratively at 365 days. The two period-specific columns come from models fitted within each period after splitting follow-up at the end of the intensive phase and later.
