# Supplementary material for: Predictors of treatment outcomes in adults with drug–sensitive Tuberculosis in Maharashtra, India: A retrospective study
Source: PLOS Glob Public Health. 2026 Nov 6;6(11):e0006539. doi: 10.1371/journal.pgph.0006539 (PMC13743022; doi:10.1371/journal.pgph.0006539)
Supplement: Supplementary file 14 [file pgph.0006539.s014.docx]

**Supporting information**

**S12_Table. Cumulative probability of death at 2 months and later from treatment initiation, mortality cohort**

| **Group** | **n** | **Deaths** | **Till 2 months, %**  **(95% CI)** | **2months to end of the year % (95% CI)** |
| --- | --- | --- | --- | --- |
| Whole cohort | 315,579 | 14,261 | 2.82 (2.76 to 2.88) | 6.25 (6.04 to 6.47) |
